# Remarks on Fractures in Ricketty Subjects

**Published:** 1846-04

**Authors:** 


*Remarks on Fractures in Ricketty Subjects.* By M. Guersant, Junior.—In children affected with rachitis, the consolidation of fractures is extremely slow; sometimes after eighteen or twenty months solid union of the fractured bones has not yet taken place; this non-consolidation is doubtless, closely connected with the existence of rickets; and this particular diathesis has not only the power of delaying the cure, but interferes also with it, during its progress by the production of some special accidents. Thus ricketty subjects are predisposed to thoracic, abdominal, and cutaneous affections. Now, when any one disease referrible to the lungs, the stomach or the skin supervenes during the treatment of a fracture, it may be safely prognosticated that the union of bone will be slow, and that the slowness of the cure will be proportioned to the intensity of the complication. Thus, if a ricketty child is affected with bronchitis or pneumonia during the treatment of a fracture, you may pronounce that the consolidation will be protracted. The prognosis will be the same if diarrhœa occurs. As to eruptive fevers, measles, and scarlatina, their unfavourable influence is even better marked than that of the above mentioned diseases. The prognosis of a fracture should, therefore, be very reserved in a case of rickets; if, however, no other complication of rachitis interferes with the formation of callus, bony union may in many cases be expected in thirty-five or forty days. The treatment of fractures in children afflicted with rachitis is essentially different in simple or compound fractures. The treatment of simple fracture should be nearly the same as that applied to healthy subjeets. Under these circumstances M. Guersant applies the common roller bandage for fracture of the femur, as follows:—M. Guersant applies a roller from the toes to the groin; three narrow splints are then placed, one on the inner side, a second, longer than the first, on the external side, and a third, on the anterior aspect of the limb. The splints should never bear upon any bony projections—a precept which gains importance by the remark, that in ricketty subjects the extremities of the long bones are almost always more voluminous than in the healthy state, and therefore more prominent. Of course, during the application of the splints, the fracture is, by the intelligent efforts of the assistants, maintained in a proper state of coaptation. The limb is then enveloped in a piece of oil cloth, in order to prevent the urine from impregnating the bandage. This precaution M. Guersant deems of paramount importance in young children, for two reasons:

—first, because it prevents the urine from irritating the skin ; and, secondly, because the renewal of the bandage is not so frequently called for. As a general rule, M. Guersant seldom finds it necessary to change the dressings more than once in a week. The application of the starch bandage to the fractures of infants is absolutely impossible. The involuntary emission of urine would constantly dissolve the starch and relax the bandage. When the patient is simply affected with rickets, M. Guersant recommends a nutritive, animal, and even stimulating diet. Some authors have asserted that feculent and farinaceous nutriment was more fitted to ricketty children than animal food ; an opinion to which M. Guersant finds an absolute answer in the fact, that the poorer orders are fed almost entirely upon beans, potatoes, and farinaceous food, and it is certainly amongst the poorer classes that rachitis is chiefly observed. M. Guersant does not recommend a sudden change in the diet of the affected subjects ; a sudden change from vegetable to animal food would, in all probability, occasion enteritis and diarrhœa ; but the child should be gradually accustomed to a more nutritious sustenance, in order that good effects may be obtained. If a complication of pneumonia or eruptive fever has occurred, it is evident that the analeptic treatment can only be instituted after the complication has been cured. The bandage should be removed very late in complicated cases. M. Gildom seldom removes it before the fortieth day ; as to medicines, he has derived benefit from the hydriodate of potass, and from the exhibition of cod-liver oil, the effects of which being very slow, are less clearly marked in hospital than in private practice.—*Medical Times.*

